# Genomic characterization of *Haemophilus influenzae*: a focus on the capsule locus

**DOI:** 10.1186/s12864-019-6145-8

**Published:** 2019-10-12

**Authors:** Caelin C. Potts, Nadav Topaz, Lorraine D. Rodriguez-Rivera, Fang Hu, How-Yi Chang, Melissa J. Whaley, Susanna Schmink, Adam C. Retchless, Alexander Chen, Edward Ramos, Gregory H. Doho, Xin Wang

**Affiliations:** 1 Bacterial Meningitis Laboratory, Meningitis and Vaccine Preventable Diseases Branch, Division of Bacterial Diseases, National Center for Immunization and Respiratory Diseases, Centers for Disease Control and Prevention, 1600 Clifton Rd NE, Mailstop H17-2, Atlanta, GA 30329 USA; 2 CDC Foundation, Atlanta, GA USA; 3 IHRC Inc., Atlanta, GA USA; 4 CSRA, Inc., Atlanta, GA USA

**Keywords:** *Haemophilus influenzae*, Whole genome sequencing, Capsule locus, Serotype, Genetic diversity, Multilocus sequence typing

## Abstract

**Background:**

*Haemophilus influenzae* (Hi) can cause invasive diseases such as meningitis, pneumonia, or sepsis. Typeable Hi includes six serotypes (a through f), each expressing a unique capsular polysaccharide. The capsule, encoded by the genes within the capsule locus, is a major virulence factor of typeable Hi. Non-typeable (NTHi) does not express capsule and is associated with invasive and non-invasive diseases.

**Methods:**

A total of 395 typeable and 293 NTHi isolates were characterized by whole genome sequencing (WGS). Phylogenetic analysis and multilocus sequence typing were used to characterize the overall genetic diversity. Pair-wise comparisons were used to evaluate the capsule loci. A WGS serotyping method was developed to predict the Hi serotype. WGS serotyping results were compared to slide agglutination (SAST) or real-time PCR (rt-PCR) serotyping.

**Results:**

Isolates of each Hi serotype clustered into one or two subclades, with each subclade being associated with a distinct sequence type (ST). NTHi isolates were genetically diverse, with seven subclades and 125 STs being detected. Regions I and III of the capsule locus were conserved among the six serotypes (≥82% nucleotide identity). In contrast, genes in Region II were less conserved, with only six gene pairs from all serotypes showing ≥56% nucleotide identity. The WGS serotyping method was 99.9% concordant with SAST and 100% concordant with rt-PCR in determining the Hi serotype.

**Conclusions:**

Genomic analysis revealed a higher degree of genetic diversity among NTHi compared to typeable Hi. The WGS serotyping method accurately predicted the Hi capsule type and can serve as an alternative method for Hi serotyping.

## Background

*Haemophilus influenzae* (Hi) can cause severe and life-threatening invasive diseases, especially in persons < 5 or ≥ 65 years old [1–3]. There are six different Hi serotypes (Hia, Hib, Hic, Hid, Hie and Hif), which each express a unique polysaccharide capsule, as well as non-typeable Hi (NTHi) strains, which lack capsule expression. Since the implementation of the Hib vaccine, the burden of Hib disease has decreased dramatically in the United States [1]. However, NTHi and other non-b serotypes continue to cause disease. For example, invasive Hia disease has exhibited more than a two-fold increase in incidence between the time periods 2002–2008 and 2009–2015 [3], highlighting the continued need to monitor the distribution of Hi serotypes.

Hi was one of the first organisms with a complete genome sequenced and its genomic characterization has provided important insights into our fundamental understanding of bacterial genomics [4–8]. The genetic diversity for Hi has predominantly been characterized through multilocus sequence typing (MLST) [9]. MLST demonstrated that each Hi serotype was associated with a few sequence types (STs), while NTHi isolates were associated with multiple STs [9–11]. Two main groups were identified: Group I contained isolates from NTHi and serotypes Hia, Hib, Hic, and Hid, while Group II contained Hia, Hie and Hif isolates [9–11]. More recently, WGS studies confirmed that NTHi isolates were more diverse, with multiple phylogenetic clades detected [12–14].

The Hi polysaccharide capsule is encoded by the capsule locus, which is composed of three regions [15]. Regions I and III are conserved among all six serotypes, while the Region II genes are unique to each serotype [15]. Region I contains the *bexABCD* operon and Region III includes the *hcsA* and *hcsB* genes, which are involved in exporting capsule polysaccharides [16–18]. Region II contains three to eight genes, depending on the serotype, and is required for polysaccharide synthesis [19–24].

Slide agglutination serotyping (SAST) and real-time PCR (rt-PCR) serotyping are commonly used to identify Hi serotypes. SAST detects polysaccharide expression using serotype-specific antibodies, while rt-PCR detects the presence of a single, serotype-specific gene [25–27]. Serotype-determination by SAST can be affected by variations in technique and the lot of antisera used [28]. Multiple reports have indicated that rt-PCR is a preferred method for serotyping because it is highly sensitive and can confirm both typeable and NTHi isolates [29, 30].

In this report, the genetic diversity of 688 Hi isolates was assessed using whole genome sequencing (WGS). In addition, a comprehensive analysis of each capsule gene was conducted to develop a WGS serotyping method to predict Hi capsule expression. Finally, the concordance between the WGS serotyping method and each of the two conventional serotyping methods, SAST or rt-PCR, was assessed in determining Hi serotypes.

## Results

### *H. influenzae* genetic diversity

**Fig. 1:**
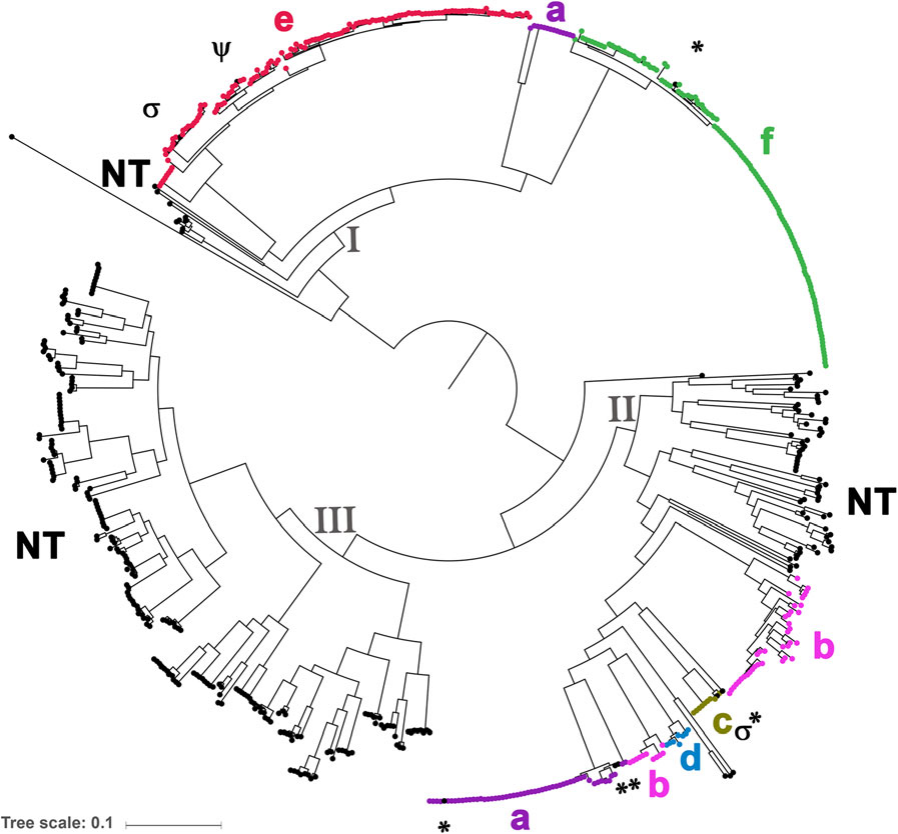
Population structure of *H. influenzae* isolates. The genomic relatedness of 688 Hi isolates is depicted as a maximum likelihood phylogeny. The three main clades (I, II, and III) are labeled on the tree and each isolate is color coded by the serotype, as determined by slide agglutination. The NTHi isolates within the serotype-specific subclades are denoted by ψ, σ, or *. The ψ indicates the isolate that had discrepant serotyping results between WGS and SAST methods. The σ indicates the only two NTHi isolates that contained an internal stop codon within a capsule gene. The * indicates the remaining five NTHi isolates (capsule nulls) that were detected within serotype-specific subclades. The tree scale is 0.1 substitutions per site along the length specified. Bootstrap values were 100/100 for all major nodes

The genetic relatedness of 688 Hi isolates was determined using a maximum likelihood phylogeny. The isolates overall clustered by serotype and formed three large clades (I, II, and III, Fig. 1). Clade I was divided into three subclades, containing predominantly Hia, Hie, or Hif isolates. Clade II contained seven subclades that contain Hia, Hib, Hic, Hid, or NTHi isolates. In contrast, clade III contained only NTHi isolates, which clustered into five separate subclades. Consistent with MLST analysis, clades II and III (Group I by MLST) were more closely related to each other than to clade I (Group II by MLST) [9, 10, 31].

A higher degree of genetic diversity was observed among NTHi than the typeable isolates. Isolates of each serotype were predominantly associated with a single subclade, with the exception of Hia and Hib. Isolates of Hia and Hib each formed two subclades. The two Hib subclades were both detected within clade II and the two Hia subclades were in clades I and II. The Hia isolates in clade II were more tightly clustered with Hib and Hid than Hic isolates, while the clade I Hia isolates were more tightly clustered with Hif than the Hie isolates. In contrast, the NTHi isolates clustered into seven separate subclades within clades II and III. The majority of NTHi isolates clustered together in clade II and III, with only eight NTHi isolates being clustered with typeable isolates within the serotype-specific subclades as denoted by *, ψ, and σ in Fig. 1.

To understand the genetic differences between the two Hia or Hib subclades, pair-wise gene comparisons were conducted to identify unique genes that distinguish between the two Hia or Hib subclades. For Hia, two genes were present in the clade I Hia isolates but absent from the isolates in clade II. An additional 57 unique genes were present in the clade II Hia isolates that were absent in the clade I isolates (Additional file 2: Table S1). For Hib, 47 genes were present in the large Hib subclade isolates but absent from all the isolates in the small Hib subclade. (Additional file 2: Table S2).

Consistent with the phylogenetic analysis, greater genetic diversity was detected among NTHi isolates by MLST, with more sequence types (STs) among NTHi compared to typeable Hi (Table 1 and Additional file 2: Table S3). Of the 284 NTHi isolates with a ST assigned, 40 % were identified as one of the 15 STs that were detected in ≥5 NTHi isolates (Table 1). In contrast, each serotype was predominantly associated with one or a few STs that differed by only one allele (Table 1). For example, the Hic, Hid, and Hif isolates were associated with ST-9, ST-10, and ST-124, respectively; the Hie isolates were associated with five genetically related STs (ST-18, ST-66, ST-121, ST-127, and ST-386). Notably, the two distinct subclades of Hia or Hib were each associated with STs that differed at all seven MLST loci. The clade I Hia isolates were ST-62 and the clade II Hia isolates were predominantly one of three genetically related STs (ST-23, ST-56 or ST-576). For Hib, the small subclade was ST-222 and the larger subclade was ST-6. Furthermore, 6/8 NTHi isolates that were detected within typeable subclades had the predominant ST associated with that subclade. For example, the two NTHi isolates in the Hie subclade were ST-66 and ST-18. A ST could not be determined for 33 Hie and 7 NTHi isolates because each isolate was missing the *fucK* gene required for the MLST analysis.

**Table 1:** MLST distribution within each serotype

Sequence Type	Number of Isolates
Hia – clade I	
ST-62	13
Other	1
Hia – clade II	
ST-23	7
ST-56	30
ST-576	10
Other	6
Hib - small clade (most similar to Hid)	
ST-222	6
Other	5
Hib - large clade	
ST-6	21
Other	17
Hic	
ST-9	7
Other	2
Hid	
ST-10	5
Other	3
Hie	
ST-18	44
ST-66	22
ST-121	8
ST-127	8
ST-386	5
ND (no *fucK* gene)	33
Other	12
Hif	
ST-124	122
Other	9
NTHi	
ST-3	13
ST-11	6
ST-12	7
ST-14	11
ST-34	6
ST-57	8
ST-103	11
ST-107	9
ST-139	8
ST-143	5
ST-145	6
ST-155	5
ST-156	8
ST-165	5
ST-182	5
ND (no *fucK* gene)	7
Other	172

Sequence types (STs) are shown for each serotype. STs detected in fewer than five isolates are grouped as “Other” and denoted in Additional file 2: Table S3. Isolates missing the *fucK* gene could not be assigned a ST and are denoted by ND (not determined).

### Genetic diversity of the *H. influenzae* capsule locus

**Fig. 2:**
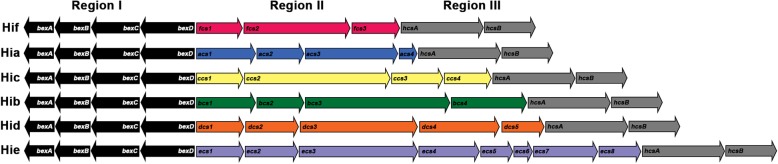
Genetic organization of the *H. influenzae* capsule genes. All serotypes contained the Region I genes *bexABCD* (black) and the Region III genes (gray): *hcsA* and *hcsB*. The Region II genes were divergent among serotypes. Each arrow represents a different gene and is labeled with the gene name. The Region II arrows are color coded by serotype

**Fig. 3:**
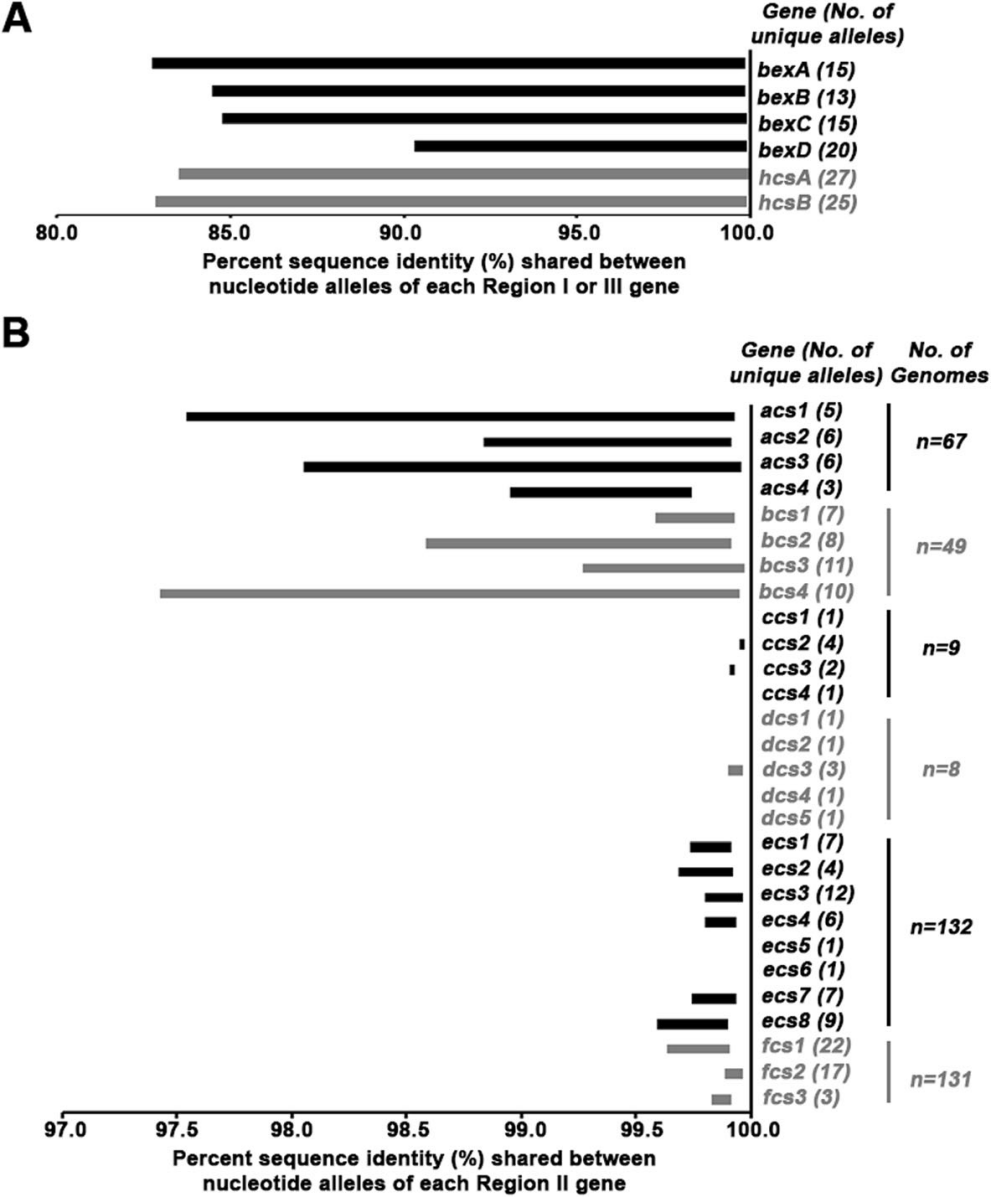
The allelic variation detected for each Region I, II and III gene. Each capsule gene from Regions I and III (**a**) or Region II (**b**) are listed on the y-axis. The unique number of alleles detected per gene is denoted within the parentheses. The number of genomes associated with that serotype is also provided. The x-axis depicts the percent identity shared among the nucleotide alleles for each capsule gene. The left side of the bar represents the minimum sequence identity and the right side represents the maximum sequence identity detected. If only one allele was detected, no data are shown

A reference database containing the alleles for each capsule gene was generated using a genome from each serotype (a total of six genomes). The reference database contained 64 alleles: six alleles for each of the six genes in Regions I and III (36 alleles total) and a single allele for each of the 28 Region II genes (Fig. 2). This reference database was queried against all 688 Hi genomes, resulting in the identification of 275 unique alleles in the three regions of the capsule locus (Fig. 3).

The allelic diversity was assessed for each capsule gene using pair-wise comparisons for all alleles detected among the 688 genomes (Fig. 3). The capsule genes in Regions I and III exhibited similar levels of sequence diversity with > 82.7% identity, except *bexD*, which was more conserved with > 90.3% identity (Fig. 3a). A gene-based, neighbor-net phylogenetic analysis demonstrated that the sequence diversity within Regions I and III was sufficient to differentiate each of the six serotypes (Additional file 1: Figure S1). The serotype-specific Region II genes exhibited high sequence similarity within each serotype, with only a few alleles detected per gene (Fig. 3b). The Region II genes of Hia and Hib exhibited a lower degree of sequence similarity compared to all other serotypes. However, if only alleles detected within the same Hia or Hib subclade were considered, the sequence similarity was more comparable to other serotypes (Additional file 1: Figure S2).

Finally, the inter-serotype sequence diversity of the Region II genes was determined using pair-wise comparisons for every possible gene pair. Only six gene pairs exhibited significant homology (Table 2). Three gene pairs (*acs2*/*bcs2*, *ccs1*/*fcs1* and *dcs5*/*ecs8*) had moderate sequence identity (< 88%) and three gene pairs (*acs1*/*bcs1*, *dcs1*/*ecs1*, and *dcs2*/*ecs2*) had high sequence identity (> 92%). The sequence identity between *acs1* and *bcs1* was comparable to the amount of allelic variation observed for *acs1* alone.

**Table 2:** Inter-serotype Sequence Identity between Region II Gene Pairs

Gene Names	Minimum Similarity	Maximum Similarity
*acs1/bcs1*	96.13	98.39
*acs2/bcs2*	56.81	62.33
*ccs1/fcs1*	85.11	85.39
*dcs1/ecs1*	92.62	92.89
*dcs2/ecs2*	94.08	94.23
*dcs5/ecs8*	86.81	87.01

### WGS serotyping is an effective method for predicting the *H. influenzae* capsule type

**Fig. 4:**
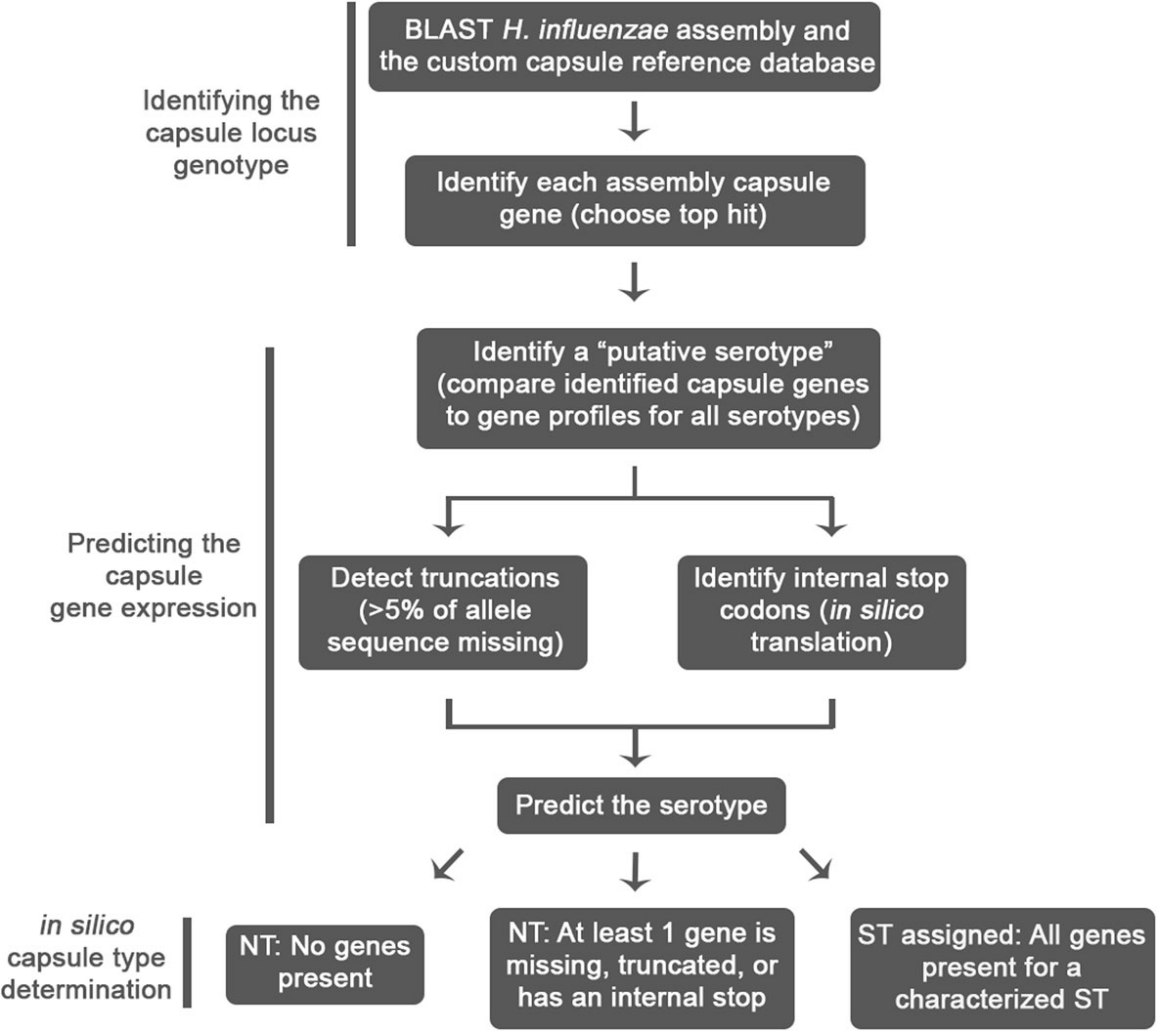
WGS serotyping method for determining *H. influenzae* serotype from WGS data. The three main steps of this process include identifying the capsule genes, predicting the capsule gene expression, and assigning the predicted serotype

An in silico method was developed to predict the Hi serotype using the following three steps (Fig. 4). First, the capsule genes within the assembly were identified using the 64 allele reference database as the query. Then, the sequence intactness of each capsule gene was assessed to predict the likelihood of expression, and finally, the prediction tool assigned a serotype. Using this method, the serotype was predicted for each of the 688 isolates, resulting in the identification of 396 typeable and 292 NTHi. Nearly all NTHi isolates (290/292) had a capsule null locus, completely lacking Region I, II and III genes. The remaining two NTHi isolates contained an internal stop codon in either *ccs2* or *ecs3* (denoted by σ in Fig. 1).

The concordance between the WGS serotyping method and SAST or rt-PCR was assessed. High concordance (99.9%) was observed between WGS and SAST in serotyping 688 isolates. One hundred percent concordance was observed between WGS and rt-PCR in serotyping 496 isolates (Table 3). Only one isolate was identified as NTHi by SAST but Hie by both WGS and rt-PCR (denoted by ψ in Fig. 1).

**Table 3:** Concordance of the WGS serotyping method and other serotyping methods

Serotype*	Concordance: SAST and WGS			Concordance: rt-PCR and WGS		
	Total Isolates	No. of Concordant Isolates	Percent	Total Isolates	No. of Concordant Isolates	Percent
a	67	67	100.0	64	64	100.0
b	49	49	100.0	46	46	100.0
c	9	9	100.0	9	9	100.0
d	8	8	100.0	8	8	100.0
e	131	131	100.0	122	122	100.0
f	131	131	100.0	131	131	100.0
NT	293	292	99.7	116	116	100.0
Total	688	687	99.9	496	496	100.0

Serotype* defined by SAST method. The concordance data is reported as the number and percent of isolates. Only one discordant isolate was identified

## Discussion

In this report, 688 invasive Hi isolates, representing all six capsule types, were characterized by WGS. The genomic analysis presented in this study was consistent with previous studies [9, 10, 12, 31]. Overall, a higher degree of genetic diversity was observed among NTHi than the typeable isolates. All 688 isolates clustered into three large clades, corresponding to Groups I and II, which were previously defined by MLST [9, 10, 31]. However, MLST may not be optimal for typing Hi because a proportion of isolates (25% of Hie and 2% of NTHi isolates) did not have the *fucK* gene required for MLST analysis. This also has important implications for rt-PCR, because *fucK* was also identified as a species-specific target for the detection of Hi [32].

Subclades associated with a specific serotype or NTHi were also identified. While no typeable isolates were observed within the NTHi-specific subclades, eight NTHi isolates were detected within serotype-specific subclades. Notably, six of the eight NTHi isolates within the serotype-specific subclades had the ST most commonly detected within the typeable isolates. It is possible that these six NTHi isolates represent strains that recently lost their capsule expression. However, capsule loss may be a rare event; otherwise one would expect to observe more NTHi in serotype specific clades.

The high resolution WGS methods used in this study provided novel insights into the genomic distinctions among the two Hia and Hib subclades. Each subclade was associated with a distinct ST and a number of unique genes. The presence of unique genes, which were further confirmed using a representative, intact genome generated by PacBio sequencing, may explain the difference between the two Hia or Hib subclades. An association between the distinct Hia and Hib subclades and specific alleles for the Region I and III genes was also observed. This finding could indicate that an ancestral strain specifically acquired the Region II genes from a Hia or Hib strain and expanded to form these subclades. However, the directionality of this potential capsule acquisition remains unclear. While the biological relevance of the two Hia or Hib subclades is still unclear, the two subclades of the same serotype had no direct association with syndromes or age.

Characterization of the capsule genes from WGS data enabled the development of an in silico method for accurately predicting the serotype, which was highly concordant with SAST and rt-PCR. Two other WGS methods for determining the Hi serotype have been recently described [14, 33]. However, this is the first comprehensive characterization of the capsule locus using a large isolate collection that includes a comparative analysis of all three serotyping methods. Consistent with the previous findings, Regions I and III are relatively conserved among all six serotypes and Region II genes are mostly unique to each Hi serotype. Interestingly, some of the Region II genes shared homology among serotypes. The sequences of *acs1* and *bcs1* were highly similar and both genes have CDP-ribitol pyrophosphorylase activity in vitro*,* which could indicate they are functionally redundant [19, 20]. Two other gene pairs (*dcs1*/*ecs1*, and *dcs2*/*ecs2)* also exhibited high sequence identity but because only a few Hid genomes were available for the study, the amount of allelic variation within each Hid gene remains unclear. Additional complementation studies are required to determine if these ORF pairs within Region II could be classified as the same gene.

High concordance was observed for the three different methods used to determine the Hi serotype. Only one discrepant isolate was detected, which was NTHi by SAST but classified as Hie by WGS and rt-PCR. There could be multiple possible explanations for this observation. This isolate could express the Hie capsule as predicted by WGS, but at an undetectable level by SAST. Alternatively, this isolate could be truly non-typeable because the expression of the polysaccharide has been disrupted by a genetic element outside the coding regions assessed by either the rt-PCR or WGS methods. Additional studies are required to determine the cause of this discrepancy. Because of the high concordance observed among the three assays used in this study, the volume of Hi isolates, the cost attributed to each assay, and the potential need for additional genomic characterization must be considered when selecting the appropriate method for serotype identification.

## Conclusions

This study provided a large-scale genomic characterization of invasive Hi isolates representing all capsule types. Whole genome phylogenetic analysis demonstrated that the isolates clustered into three large clades, with typeable isolates forming distinct, serotype-specific subclades. The in-depth characterization of the capsule locus highlighted that Regions I and III are highly conserved among serotypes, while very little homology was observed between Region II genes. Finally, a novel WGS method was developed to determine the Hi serotype, demonstrating that the capsule gene content is a strong and accurate predictor of capsule expression.

## Methods

### *H. influenzae* culture and serotyping

Hi isolates were collected from 1990 to 2017 through surveillance programs. A total of 675 isolates were collected from 27 U.S. states and 13 isolates were obtained from three different countries. All serotypes were represented: Hia *n* = 67, Hib *n* = 49, Hic *n* = 9, Hid *n* = 8, Hie *n* = 131, Hif *n* = 131, NTHi *n* = 293. Hi isolates were cultured on chocolate II agar with hemoglobin and IsoVitaleX (BD BBL) at 37 °C with 5% CO_2_. The serotype was determined by SAST using serotype-specific antisera obtained from Remel and by rt-PCR as described previously [28, 34, 35].

### Whole genome sequencing and phylogenetic analysis

DNA was extracted manually using the Gentra Puregene yeast/bacteria DNA extraction kit (Qiagen) or with a chemagic Prepito instrument (PerkinElmer) using the Cyto Pure Kit. Genomic libraries were generated using the NEBNext Ultra DNA kit according to manufacturer instructions. Sequencing was completed on a MiSeq or HiSeq 2500 using 250 bp paired-end reads. Raw reads were filtered and trimmed using cutadapt, version 1.8.1 [36], and assembled into genomes using SPAdes, version 3.7.0 [37]. A subset of genomes were generated using PacBio methods as described previously [38]. The dataset supporting the conclusions of this article is available in the NCBI repository, [Bioproject: PRJNA512636].

The maximum likelihood phylogenetic tree was generated using whole genome data and kSNP3 [39]. The tree was annotated as graphics using the iTol program [40]. All genes within each assembly were identified by comparing the sequences against the PubMLST *H. influenzae* collection [41]. The neighbor-net phylogenetic network for the Region I and III capsule genes was generated using SplitsTree4 after aligning the genes with MUSCLE [42, 43]. BLAST was used to identify the MLST loci present within each assembly; allele numbers and STs were assigned with PubMLST [41, 44]. To identify the genes that distinguished the two subclades for Hia or Hib, gene by gene comparisons were conducted and gene function was assigned using InterProScan 5 [45]. The presence or absence of genes among isolates from each subclade were compared using a custom in-house python script. In addition, all reported, differentially detected genes were manually curated to confirm the absence of any BLAST results within a representative PacBio assembly from that subclade.

### Characterization of the *H. influenzae* capsule locus genes

A reference database containing at least one allele for each Hi capsule gene was generated. Reciprocal best hits (BLAST) was used to identify the capsule locus alleles within a GenBank genome sequence for each serotype: Hia (CP077811), Hib (FQ312006), Hic (HQ651151) [21], Hid (HQ424464) [21], Hie (FM882247) [22], and Hif (CP005967). Additional refinement was completed by manually curating the start and stop sites of each open reading frame. All of the capsule alleles identified through this process were compiled into a custom reference database. BLAST was used to identify the capsule alleles within the 688 isolate genomes using the custom reference database as a query.

To assess the homology among genes within the capsule locus, pair-wise comparisons were completed using BLAST and the percent sequence identity was reported [44]. If genes varied in length, the longer allele was used as the query sequence. To determine the allelic sequence diversity, all identified alleles for each Region I, II or III gene were compared. For inter-serotype comparisons, every possible Region II gene pair, across all serotypes, was assessed.

### Prediction of *H. influenzae* serotype from WGS data

Each genome assembly was compared against the custom reference database using BLAST [44]. All BLAST hits with 90% or greater identity to an allele in the reference database were pooled. If two genes matched the same region of the genome, the gene with the lower alignment score (defined as (Identity*Alignment Length)/Allele Length) was discarded to ensure preference was given to exact matches rather than longer, inexact matches. To detect potential internal stop codons, in silico translations were completed using the BioPython Seq module [46].

To predict capsule type, the top hits for each assembly were compared to the known essential genes for each serotype. The capsule genotype was assigned based on the highest number of corresponding genes identified. To predict the capsule expression, factors that could impact expression were considered: missing genes, premature internal stops, and truncated genes (those that had 95% or less coverage when aligned against its closest reference). If all genes were present and intact, the serotype was predicted to be the serotype with the highest number of identified genes. Non-typeable (NTHi) was assigned to assemblies containing internal stops, truncated or missing capsule genes. The capsule prediction method related to this project has been made publicly available (https://github.com/ntopaz/hinfluenzae_capsule_characterization).

### Assessing concordance among three serotyping assays

Concordance between SAST and WGS was calculated using all 688 isolates; concordance between rt-PCR and WGS was assessed using the 496 isolates (Hia *n* = 64, Hib *n* = 46, Hic *n* = 9, Hid *n* = 8, Hie *n* = 123, Hif *n* = 131, NTHi *n* = 115 as defined by SAST methods) that had been tested by rt-PCR. Any isolate identified to have a discrepant result was re-tested by the discrepant method for confirmation. Only confirmed discrepant isolates were included.

## Supplementary information

**Additional file 1: Figure S1.** Neighbor-net phylogenetic analysis of the Region I and III alleles. A gene-based neighbor-net phylogenetic analysis was generated by aligning the alleles of the Region I and III genes. The sequence diversity present within the Region I and III genes was sufficient to differentiate the isolates from each of the six serotypes, including the distinct Hia and Hib subclades. **Figure S2.** The allelic variation detected within and between subclades for Hia and Hib Region II genes. The x-axis depicts the percent identity shared among alleles for each capsule gene. The left side of the bar represents the minimum sequence identity and the right side represents the maximum sequence identity detected. Allelic variation was quantified for either alleles detected within the same subclade (intra-subclade) or between the two subclades (inter-subclade).

**Additional file 2: Tables S1 and S2.** Genes differentially detected between the two Hia subclades (Table S1) or the two Hib subclades (Table S2). A value of 1 indicates 100% of the isolates in that subclade contained the gene; a value of 0 indicates that the gene was detected in 0% of isolates within that subclade. **Table S3.** Sequence types detected in < 5 isolates. ND = Not determined.

## Data Availability

The dataset generated during the current study is available in the NCBI repository, [Bioproject: PRJNA512636]. The capsule prediction method related to this project has been made publicly available (https://github.com/ntopaz/hinfluenzae_capsule_characterization).

## Abbreviations

Hi: *Haemophilus influenzae*
Hia: *Haemophilus influenzae* serotype a
Hib: *Haemophilus influenzae* serotype b
Hic: *Haemophilus influenzae* serotype c
Hid: *Haemophilus influenzae* serotype d
Hie: *Haemophilus influenzae* serotype e
Hif: *Haemophilus influenzae* serotype f
MLST: Multilocus sequence typing
NTHi: Non-typeable *Haemophilus influenzae*
rt-PCR: Real-time polymerase chain reaction
SAST: Slide agglutination serotyping
ST: Sequence type
WGS: Whole genome sequencing
