# Supplementary material for: Genomic characterization of Haemophilus influenzae: a focus on the capsule locus
Source: BMC Genomics. 2019 Oct 12;20:733. doi: 10.1186/s12864-019-6145-8 (PMC6790013; doi:10.1186/s12864-019-6145-8)
Supplement: Supplementary file 1 — Additional file 1: Figure S1. Neighbor-net phylogenetic analysis of the Region I and III alleles. A gene-based neighbor-net phylogenetic analysis was generated by aligning the alleles of the Region I and III genes. The sequence diversity present within the Region I and III genes was sufficient to differentiate the isolates from each of the six serotypes, including the distinct Hia and Hib subclades. Figure S2. The allelic variation detected within and between subclades for Hia and Hib Region II genes. The x-axis depicts the percent identity shared among alleles for each capsule gene. The left side of the bar represents the minimum sequence identity and the right side represents the maximum sequence identity detected. Allelic variation was quantified for either alleles detected within the same subclade (intra-subclade) or between the two subclades (inter-subclade). [file 12864_2019_6145_MOESM1_ESM.docx]

**SUPPLEMENTAL FIGURE 1**


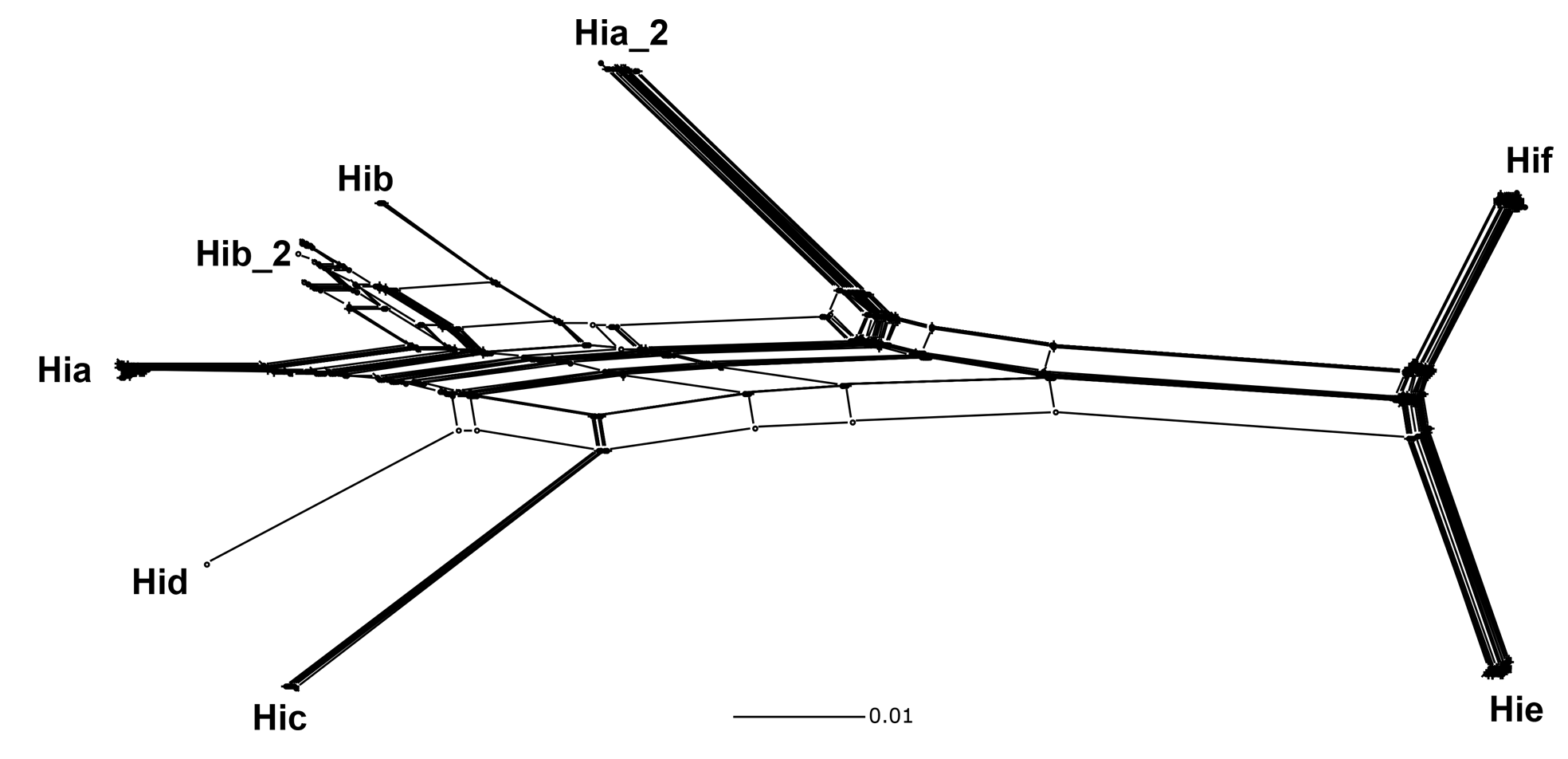


**Figure S1. Neighbor-net phylogenetic analysis of the Region I and III alleles.** A gene-based neighbor-net phylogenetic analysis was generated by aligning the alleles of the Region I and III genes. The sequence diversity present within the Region I and III genes was sufficient to differentiate the isolates from each of the six serotypes, including the distinct Hia and Hib subclades.

**SUPPLEMENTAL FIGURE 2**


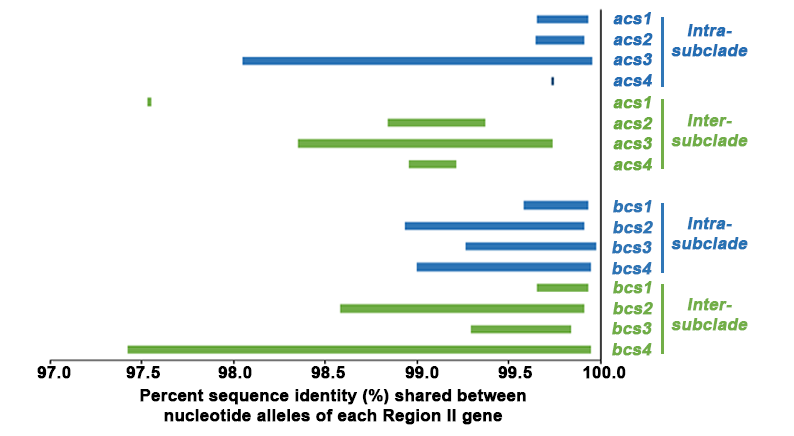


**Figure S2. The allelic variation detected within and between subclades for Hia and Hib Region II genes.** The x-axis depicts the percent identity shared among alleles for each capsule gene. The left side of the bar represents the minimum sequence identity and the right side represents the maximum sequence identity detected. Allelic variation was quantified for either alleles detected within the same subclade (intra-subclade) or between the two subclades (inter-subclade).
